# Economic Migrants and Clinical Course of SARS-CoV-2 Infection: A Follow-Up Study

**DOI:** 10.3389/ijph.2022.1605481

**Published:** 2022-12-16

**Authors:** Julia Martínez-Alfonso, Arthur Eumann Mesas, Nuria Jimenez-Olivas, Antonio Cabrera-Majada, Vicente Martínez-Vizcaíno, José Manuel Díaz-Olalla

**Affiliations:** ^1^ Primary Care University Center Daroca, Madrid, Spain; ^2^ Universidad de Castilla-La Mancha, Cuenca, Spain; ^3^ Postgraduate Program in Public Health, Universidade Estadual de Londrina, Londrina, Brazil; ^4^ Facultad de Ciencias de la Salud, Universidad Autónoma de Chile, Talca, Chile; ^5^ Madrid Salud Public Health Institute of Madrid, Madrid, Spain

**Keywords:** COVID-19, social determinansts of health, migrant healthcare, hospitalization, follow up

## Abstract

**Objective:** To analyze whether social deprivation and economic migrant (EM) status influence the risk of being hospitalized with COVID-19.

**Methods:** This was a retrospective follow-up study including all patients older than 18 years attending the Daroca Health Center in Madrid, Spain, diagnosed with COVID-19 during September 2020. Data on EM status and other sociodemographic, lifestyle and comorbidities that could affect the clinical course of the infection were obtained from electronic medical records.

**Results:** Of the 796 patients positive for COVID-19, 44 (5.53%) were hospitalized. No significant differences were observed between those who were hospitalized and those who were not in the mean of social deprivation index or socioeconomic status, but EM status was associated with the risk of being hospitalized (*p* = 0.028). Logistic regression models showed that years of age (OR = 1.07; 95% CI: 1.04–1.10), EM status (OR = 5.72; 95% CI: 2.56–12.63) and hypertension (OR = 2.22; 95% CI: 1.01–4.85) were the only predictors of hospitalization.

**Conclusion:** Our data support that EM status, rather than economic deprivation, is the socioeconomic factor associated with the probability of hospital admission for COVID-19 in Madrid, Spain.

## Introduction

The COVID-19 pandemic is affecting worldwide populations, but although everyone is susceptible to being infected by the virus, the risk of acquiring the disease is higher in population groups with lower socioeconomic status (SES) [1, 2]. Similarly, it appears that the consequences of SARS-CoV-2 infection are conditioned, in addition to clinical parameters [3–5], by socioeconomic determinants and the patient’s environment [6].

In most countries, COVID-19 has disproportionately affected poor and excluded populations, who have a higher prevalence of chronic diseases, which, in turn, are risk factors for acquiring COVID-19. Social determinants of health (SDH), including SES, neighborhood and race or ethnicity, may have a significant role in COVID-19 outcomes because they can influence the probability of developing these chronic diseases [7] and also of receiving adequate management and treatment for them and for COVID-19 [8–11]. In Spain, due to the universal free access to the health system, the quality of healthcare may not be decreased in these disadvantaged populations, but their accessibility may be more limited for several causes, including work-related reasons, health system overload or ignorance of access mechanisms [12]. However, whether there have been inequalities by SES in the access to COVID-19 diagnostic tests and whether SES has influenced the severity of the clinical course of SARS-CoV-2 infections remain unknown, although it has been suggested that there may have been a relationship between EM status and SES with the risk of being infected and hospitalized [13].

Similarly, a study using UK Biobank data reported a marked increase in the risk of hospitalization for COVID-19 as a function of race and social deprivation [14]. Similarly, another UK study reported a strong association between social deprivation and the risk of death from COVID-19, which was only partially attributable to the presence of comorbidities [4]. Additionally, in France, people living in the most deprived areas have a higher risk of testing positive for SARS-CoV-2 [15].

Migration is one of the SDH [16] that can affect both disease profiles and access to preventive measures and treatment, and poses a threat to the physical and mental health of individuals, placing them in a situation of vulnerability [17]. The expression economic migrant (EM) refeers to people who migrate due to economic hardship or natural disasters often suffer several inequalities in relation to the native population, such as worse job and housing opportunities and lack of social support and discrimination. In addition, even countries with universal healthcare systems, such as Spain, may face barriers in accessing health services [12] due to lack of knowledge of the system, incompatibility of schedules, job insecurity or economic difficulties in terms of transport and financing of treatment [18].

Although consistent evidence coming from large databases from European countries is available [15, 19–21], studies using primary data from primary care services are scarce [22, 23]. To our knowledge, no study in Spain has jointly analyzed the importance of social deprivation and economic migration in the clinical course of SARS-CoV-2 infection. Thus, this study aimed to jointly analyze whether social deprivation and EM status influence the risk of being hospitalized with COVID-19 and the clinical course of the disease after hospitalization in Madrid, Spain.

## Methods

### Design, Setting and Participants

This was a retrospective follow-up study including all patients with 18 years of age and older who were diagnosed with COVID-19 through PCR tests from 31 August to 29 September 2020 at the Daroca Health Center in Madrid, Spain. During this period, 796 patients with at least one positive RT‒PCR test by nasopharyngeal exudate analysis were registered in this health center out of a total of 2606 tests performed.

### Measures

- Economic migrant status. People born in a low-income country were considered EM, irrespective of their nationality. Low-income countries exclude Spain, Austria, Belgium, Cyprus, Denmark, Finland, France, Greece, Ireland, Iceland, Italy, Liechtenstein, Luxembourg, Malta, Monaco, Norway, the Netherlands, Portugal, Andorra, the United Kingdom, Germany, San Marino, the Vatican, Sweden, Switzerland, Canada, the United States of America, Japan, Australia and New Zealand [24].
- Deprivation index by census section (DI). This index is calculated for each census section and was collected from the 2021 Madrid City Health Study [25]. It combines information about the following variables: percentage of people aged 30–64 with secondary education or less, registered unemployment rate between the ages of 16 and 65, percentage of economic migrants, *per capita* incomes and the percentage of people who abstained from voting the last assembly of Autonomous Community of Madrid elections. The census section was obtained from the address details (street, number) of participants. This index was categorized into quintiles, with the fourth and fifth quintiles considered low SES and the first, second and third quintiles considered medium-high SES.
- Clinical factors associated with risk of death from COVID. We used electronic medical records from the primary healthcare system to gather data from the following variables and health conditions: age, sex, weight status, smoking, diabetes, recently diagnosed (less than 5 years) or active cancer, hypertension, cardiovascular disease, chronic pulmonary disease and chronic kidney disease.

#### Outcome Variables

##### Primary: Admission to Hospital

Secondary outcome variables: length of hospital stay, need for high flow oxygen therapy during admission, need for bolus steroid therapy during admission, admission to intensive care unit (ICU) or intermediate respiratory care unit (IRCU) and an indicator of poor disease progression that includes data about length of hospital stay, admission to ICU/IRCU or death. They were collected by physician investigators from the hospital electronic health record.

### Ethical/Legal Issues

This study was approved by the Ethical Committee for Clinical Research of the Hospital de la Princesa. The data of this study were kept under the custody of the principal investigator, who is responsible for maintaining their confidentiality.

### Statistical Analysis

#### Statistical Power

Calculated using GRANMO software (https://www.imim.cat/ofertadeserveis/software-public/granmo/), for bilateral hypothesis tests, assuming differences in the hospital admission rates between EM and the native population of 3.3%, an alpha error of 0.05 and a beta error of 0.2, we required a sample size of 44 participants in both the exposed group and the non-exposed group for detecting a minimum relative risk of 0.3. This sample size, while reasonable for estimating differences between the EM and native population, was not sufficient for regression models with a large number of covariates, as in our case, since it is estimated that approximately 15 (hospitalized) cases are needed for each covariate [26]. This is why we considered it necessary to estimate a joint coefficient (propensity score) [27] for the set of comorbidity covariates that were not of interest for our analysis to gain statistical power.

#### Data Analyses

The results are presented as frequencies and proportions for categorical variables and means (SDs) for quantitative variables. Mean age and DI differences between those who were admitted to the hospital and those who were not were examined using Student’s t-test. Likewise, Pearson’s chi-squared test was used to test the hypothesis of an association between sex, smoking, immunosuppression, EM status and comorbidities (when any cell showed expected values less than 5, Fisher’s exact test was used as a hypothesis test) and the probability of being hospitalized or not.

Because no differences were found in the mean DI between participants who were hospitalized and those who did not and considering that EM status was the only socioeconomic indicator that was associated in bivariate analysis with the risk of hospitalization, we examined the differences by EM status in age, deprivation index, immunosuppression, smoking and comorbidities in both hospitalized and non-hospitalized patients. In those hospitalized, we also examined the mean duration of admission, the proportion of subjects who received high-flow oxygen therapy, steroid boluses and who were admitted to the ICU/IRCU by EM status (Table 2).

Among subjects hospitalized by COVID-19, as a close surrogate for in-hospital disease outcome, subjects were considered to have a poor outcome if they died in the hospital, required admission to the ICU/IRCU or if the length of stay was equal to or longer than the median hospital stay of the subjects in the sample; all those who did not meet any of these characteristics were considered to have a good outcome. Student’s t-test for independent samples was used to test the hypothesis of differences in mean length of stay, age and deprivation index by good/poor in-hospital outcomes.

Binary logistic regression models were used to estimate the main predictors of hospitalization (deprivation index, age, sex, immigrant status, smoking, immunosuppression and the different comorbidities). Due to the lack of statistical power for the analysis of predictors of hospital admission and in view of the fact that the main predictor of hospital admission, in addition to age, was EM status, propensity scores were calculated for this condition (0 = no EM, 1 = EM), with sex, deprivation index, smoking, immunosuppression and comorbidities as predictors (variables showing an association in the full regression model *p*-value ≤0.30). Methods based on propensity scores allow us to combine several confounders into a single variable (the propensity score). Therefore, propensity scores reduce the number of independent covariates to be included in statistical models by synthesizing a set of covariates into a single variable [27].

Thus, we estimated logistic regression models with hospital admission as the dependent variable and the variables showing an association in the full regression model *p*-value ≤0.30 as independent variables (unadjusted Table 4) and, as a sensitivity analysis, adjusted for propensity scores (Table 5). In the first case, we used stepwise procedures (Wald method) for selecting predictors, and in the second case, we used age, immigrant status, obesity, hypertension and propensity score as predictor variables.

Statistical analysis was performed with IBM SPSS Statistics version 28, except for the propensity scores, which were calculated with STATA version 16.

## Results

In total, during September 2020, in the Daroca Primary Care Health Centre, 796 patients older than 17 years (55.5% women) were diagnosed with COVID-19 by PCR. Of them, 44 (5.5%) were hospitalized. The mean age was higher (*p*-value <0.05) in those hospitalized (61.75 years; SD = 15.78 years) than in those not hospitalized (45.53 years; SD 16.57 years). Forty-one percent of the subjects were EM, with a significantly higher proportion in the hospitalized (56.8%) than in the non-hospitalized (40.0%). No significant differences were observed between hospitalized and non-hospitalized subjects in the mean DI (Table 1).

**TABLE 1:** Clinical and sociodemographic characteristics of the study sample according to the hospitalization status. Economic migrants and clinical course of SARS-CoV-2 infection: a follow-up study, Madrid, Spain, 2020.

Variable	Total (n = 796)	Hospitalization		*p*-value
		Yes (*n* = 44)	No (*n* = 752)	
Age, years	46.42 ± 16.93	61.75 ± 15.78	45.53 ± 16.57	0.001
Female sex, n (%)	450 (56.50)	26 (59.10)	424 (56.40)	0.725
Deprivation index, score	0.45 ± 0.07	0.46 ± 0.07	0.45 ± 0.07	0.455
Economic migrant, n (%)	326 (41.00)	25 (56.8)	301 (40.0)	0.028
Obesity, n (%)	126 (15.80)	14 (31.82)	112 (14.89)	0.003
Tobacco, n (%)				0.012
Smoker, n (%)	103 (12.90)	1 (2.30)	102 (13.60)	
Ex-smoker, n (%)	60 (7.50)	7 (15.90)	53 (7.00)	
Hypertension, n (%)	138 (17.30)	21 (47.70)	117 (15.60)	0.001
Diabetes, n (%)	68 (8.50)	10 (22.70)	58 (7.70)	0.001
Immunosuppression, n (%)	17 (2.10)	0 (0)	17 (2.30)	0.313
Active cancer or treated and in complete remission within the last 5 years, n (%)	12 (1.60)	1 (2.30)	11 (1.50)	0.224
Cardiovascular disease, n (%)	26 (3.30)	3 (6.80)	23 (3.10)	0.173
Chronic kidney disease, n (%)	23 (2.90)	6 (13.60)	17 (2.30)	0.001
Chronic pulmonary disease, n (%)	37 (4.60)	4 (9.10)	33 (4.40)	0.150

Values are means ± standard deviations (or number of participants and percentage when indicated).

Table 2 presents the characteristics of hospitalized and non-hospitalized individuals by EM status. Overall, the EM that were not hospitalized were significantly younger, had a higher mean DI, and had a lower prevalence of smokers and people with diabetes, hypertension, cardiovascular disease and chronic pulmonary and kidney disease. Hospitalized EM people were not significantly different in any of the variables studied, except that they were almost 20 years younger, and their prevalence of hypertension and cardiovascular disease was significantly lower (*p* < 0.05). In terms of treatment during hospital admission, a higher proportion of non EMs received high-flow oxygen therapy and corticosteroid boluses, and a higher proportion were admitted to intensive care, although all these differences did not reach statistical significance. Moreover, in terms of clinical evolution among hospitalized patients (Table 2), apart from the length of stay, no variable showed significant differences.

**TABLE 2:** Clinical and sociodemographic characteristics of non-hospitalized and hospitalized people with COVID-19 diagnosis according their economic migrant status. Economic migrants and clinical course of SARS-CoV-2 infection: a follow-up study, Madrid, Spain, 2020.

Variable	Not hospitalized (*n* = 752)			Hospitalized (*n* = 44)		
	Non-immigrant	Immigrant	*p*-value	Non-immigrant	Immigrant	*p*-value
Age, years	47.79 ± 18.6	42.14 ± 12.2	0.001	72.95 ± 13.8	53.24 ± 11.3	0.001
Deprivation Index, score	0.4 ± 0.7	0.46 ± 0.7	0.001	0.46 ± 0.7	0.45 ± 0.7	0.758
Obesity, n (%)	66 (14.6)	46 (15.3)	0.807	6 (31.6)	8 (32)	0.976
Tobacco			0.002			0.509
Smoker, n (%)	70 (15.5)	32 (10.6)		1 (5.3)	—	
Ex-smoker, n (%)	41 (9.1)	12 (4)		3 (15.8)	4 (16)	
Diabetes, n (%)	42 (9.3)	16 (5.3)	0.044	6 (31.6)	4 (16)	0.222
Hypertension, n (%)	92 (20.4)	25 (8.3)	0.001	14 (73.7)	7 (28)	0.003
Active cancer or treated and in complete remission within the last 5 years, n (%)	9 (2)	2 (0.7)	0.136	1 (5.3)	—	0.246
Immunosuppression, n (%)	10 (2.2)	7 (2.3)	0.922	—	—	
Cardiovascular disease, n (%)	19 (4.2)	4 (1.3)	0.024	3 (15.8)	—	0.040
Chronic pulmonary disease, n (%)	26 (5.8)	7 (2.3)	0.024	1 (5.3)	3 (12)	0.441
Chronic kidney disease, n (%)	17 (3.8)	—	0.001	5 (26.3)	1 (4)	0.033
Mean length of hospital stay, days				11.37 ± 8.8	11.68 ± 18.8	0.947
High-flow oxygen therapy, n (%)				5 (26.3)	9 (36)	0.495
Bolus steroid therapy, n (%)				15 (78.9)	24 (96)	0.077
Intensive Care Unit admission, n (%)				2 (10.5)	6 (24)	0.251

Values are means ± standard deviations (or number of participants and percentage when indicated).

Logistic regression models including all the potential predictor variables showed that the only risk factors for being admitted to the hospital in patients with COVID-19 were age and EM status (Table 3). Of note, the probability of hospital admission increased by 7% for each additional year of age (OR = 1.07). However, in a forward stepwise model (Table 4), age, EM status and hypertension showed significant predictive ability.

**TABLE 3:** Binary logistic regression model estimating the association between clinical and sociodemographic variables and hospitalization risk in patients with COVID-19. Economic migrants and clinical course of SARS-CoV-2 infection: a follow-up study, Madrid, Spain, 2020.

Variable	B	Standard error	Wald	*p*-value	Odds ratio
Age (years)	0.064	0.014	19.692	<0.001	1.066
Female sex (yes, no)	0.194	0.358	0.294	0.587	1.215
Immigrant status (yes, no)	1.896	0.435	18.972	<0.001	6.657
Obesity (yes, no)	0.430	0.382	1.270	0.260	1.537
Deprivation Index (score)	0.186	2.358	0.006	0.937	1.204
Tobacco (yes, no)	−0.023	0.272	0.007	0.934	0.978
Diabetes (yes, no)	0.216	0.466	0.214	0.643	1.241
Hypertension (yes, no)	0.708	0.432	2.683	0.101	2.030
Active cancer or treated and in complete remission within the last 5 years	0.005	0.227	0.001	0.981	1.005
Immunosuppression (yes, no)	−18.942	8669.528	0.000	0.998	0.000
Cardiovascular disease (yes, no)	−0.543	0.730	0.552	0.457	0.581
Chronic pulmonary disease (yes, no)	0.088	0.626	0.020	0.888	1.092
Chronic kidney disease (yes, no)	0.558	0.624	0.799	0.371	1.747

**TABLE 4:** Predictors of hospitalization in patients with COVID-19 in a logistic regression model (forward selection strategy, Wald method) controlling for admission predictive variables in a forward stepwise model. Economic migrants and clinical course of SARS-CoV-2 infection: a follow-up study, Madrid, Spain, 2020.

Variable	B	Standard error	Wald	*p*-value	Odds ratio
Age (years)	0.064	0.013	22.975	<0.001	1.066
Immigrant status (yes, no)	1.744	0.404	18.666	<0.001	5.723
Hypertension (yes, no)	0.796	0.400	3.972	0.046	2.217

Variables that did not meet statistical criteria to be included in the model: obesity, diabetes, immunosuppression, tobacco, active cancer or treated and in complete remission within the last 5 years, cardiovascular disease, chronic kidney disease and chronic pulmonary disease. B, beta coefficient.

Last, when as a sensitivity analysis, in addition to the variables with *p* < 0.3 in the full logistic regression model in Table 3, a logistic regression model was estimated with the inclusion of a propensity score calculated from lifestyle, socioeconomic and comorbidity variables that could presumably be associated with EM status (Table 5) was introduced. It was observed that the only variables with a significant OR were EM status (OR = 10.72), hypertension (OR = 3.28) and age (OR = 1.08). All first-order interaction terms were tested, and none was significant.

**TABLE 5:** Binary logistic regression model including the main hospital admission predictors adjusting for propensity scores^a^. Economic migrants and clinical course of SARS-CoV-2 infection: a follow-up study, Madrid, Spain, 2020.

Variable	B	Standard error	Wald	*p*-value	Odds ratio
Age (years)	0.081	0.019	18.728	<0.001	1.085
Immigrant status (yes, no)	2.371	0.620	14.645	<0.001	10.708
Obesity (yes, no)	0.571	0.381	2.249	0.134	1.771
Hypertension (yes, no)	1.188	0.514	5.339	0.021	3.279
Propensity score	−4.541	3.242	1.962	0.161	0.011

^a^ The propensity score was calculated using migrant status as a dependent variable and sex, deprivation index, tobacco, diabetes, active cancer or treated and in complete remission within the last 5 years, immunosuppression, cardiovascular disease, chronic pulmonary disease and chronic kidney disease. B, beta coefficient.

## Discussion

The present study is, to our knowledge, the first to jointly analyze, in a Spanish population, the influence of a social deprivation index as an indirect measure of family SES and EM status on the probability of being hospitalized for COVID-19. Our data support that, contrary to expectations, socioeconomic deprivation is not a significant predictor of hospital admission. However, EM status was found to be, along with age and hypertension, an important predictor of hospital admission. Our data also suggest that obesity, as reported by some authors [28], could also be a predictor of hospital admission for COVID-19. However, the results from our study do not allow to draw a firm conclusion as obesity did not reach statistical significance, perhaps due to the small sample size. Additionally, this lack of statistical power did not allow us to assess predictors of clinical course as intended.

The relationship between living conditions associated with socioeconomic deprivation and COVID-19 hospitalization rates is a controversial issue. Thus, a study in Switzerland reported that people living in low socioeconomic neighborhoods were less likely to be tested for SARS-CoV-2 infection but more likely to test positive and be admitted to hospital or die than those living in less deprived areas [29]. Similarly, in several geographic areas of the United States, a strong association between living in a disadvantaged area and the risk of getting SARS-CoV-2 infection and of being hospitalized for SARS-CoV-2 infection has been reported [30]. On the other hand, a study in San Francisco reported a relationship between social vulnerability and COVID-19 hospitalization rates, although this study noted that COVID-19 outcomes were not systematically explained by increased social vulnerability, as this relationship occurred only in white people but not in those of other ethnic groups [31]. Our data do not support that living in an area of higher social deprivation increases the likelihood of being hospitalized for COVID-19. In fact, no significant differences were observed between the mean DI of patients with SARS-CoV-2 infection who were hospitalized and those who were not, nor were they observed when DI was categorized into high and low SES. Several reasons can be proposed to justify this apparent discrepancy with the majority of studies published thus far. Among them, the patients in our study came from a fairly socioeconomically disadvantaged area. Another possible reason that needs to be taken into account when analyzing our data is a possible ecological fallacy, since the DI calculated for census units, and the neighborhood in general, is a population measure, and our inferences are individual. Although much of the literature is based on data similar to ours, a multilevel analysis, something that our small number of hospitalizations does not allow, would be the appropriate procedure to distinguish these two levels of aggregation.

Studies conducted in Spain [13, 32, 33] and in various countries around the world [20, 34] coincide in indicating worse consequences of SARS-CoV-2 infection, including hospitalization, in migrants than in the host population, although there is great variability in the strength of this association [13]. In our study, in addition to a higher infection rate (in our sample including all PCR-positive individuals, there was 41% EM, while in the district from which the sample was drawn, 31% of individuals were born outside Spain), a strong association was observed between EM status and the probability of hospitalization for COVID-19. Despite this high rate of infection in EMs already described in other studies [13], it is possible that this group has a higher proportion of unconfirmed cases due to difficulties in access and other circumstances associated with EM status and that this ultimately leads to an overestimation of the hospitalization rate in this group. Even so, in the logistic regression analyses, in addition to age, EM status was found to be the factor most closely related to hospitalization, such that EMs are five times more likely to be hospitalized for the infection than the native population; moreover, this association holds after adjusting for age, hypertension, obesity and other pathologies (the latter synthesised in a propensity score).

Many of the hypotheses that could be suggested to explain why EMs are more likely to be hospitalized than the native population, such as population density at the place of residence or other indicators of socioeconomic deprivation [13, 20, 34] are not applicable in our case, as the DI shows no significant differences between immigrants and natives. Likewise, the higher hospitalization rates in EM would apparently not be in accordance with the phenomenon known as the “healthy immigrant effect” [35], although this gap in health levels between EM and natives has been questioned in Spain and other European countries [36]; moreover, controlling for comorbidity using propensity scores also raises the suspicion that it is not associated pathologies that increase the probability of hospitalization in EMs. Therefore, given that comorbidity, socioeconomic and demographic factors cannot be identified as responsible for the association between EM status and the risk of being hospitalized for COVID-19, one can only speculate, as has been recently suggested [37], whether language and cultural barriers, lack of knowledge about the functioning of the health system, sociopolitical conditioning factors or genetic and biological susceptibility might be behind these differences.

The main limitation of this study is the lack of statistical power due to the small number of hospital admissions in the sample, which does not allow us to make solid estimates of the risk of poor outcomes by socioeconomic level, deprivation index, and each of the important comorbidities, as was intended in this study. Similarly, although it has been reported that the rate of hospitalization is associated with multiple pathologies [38], our study only allowed us to clearly establish that hypertension was a risk factor for hospitalization, while it was not possible to establish whether obesity also contributes to an increased risk of hospitalization, something that is in question [28]. In this sense, the fact of not using primary data but data obtained from medical records could also limit the precision of our estimates. On the other hand, our data are threatened by a potential selection bias because an unknown proportion of cases did not go to the Health Centre for diagnosis but were probably confined by their own decision in the face of suggestive symptomatology, and we cannot know the influence of this bias on our estimates; furthermore, other potential selection biases could correspond to those patients who, because they do not have public healthcare for reasons of social inequities, are not represented in our sample and whose influence on our estimates is difficult to predict. Likewise, another potential selection bias could be related to those patients who have not been included because they did not go to the Health Centre for the PCR test but to other private or public centers. Finally, as noted above, the DI is not a variable that reflects individual SES but that of the group of people living in the same census tract. Theoretically, therefore, the analysis in this study should have used hierarchical models that take into account individual variables and census cluster variables, which would have greatly increased the sample size needed. However, by reflecting all the variables in a single index (DICS), we consider this as an individual variable in the analysis, in the same way as is usually done with household SES, which is often treated as an individual variable, when in fact it is a cluster variable. In any case, in view of the results, DICS did not play a significant predictive role, perhaps because of the shortcomings of this index for making individual inferences.

### Conclusion

Our data support that economic deprivation, unlike EM status, does not play a significant role in the probability of hospital admission for COVID-19. Furthermore, and considering the disparity of cultural, political and social factors behind the association between immigration and the probability of being infected and hospitalized by COVID-19, multidisciplinary studies are needed to investigate the complex mechanisms underlying the association between EM and the risk of being hospitalized by COVID-19 and the potential health inequities derived from it. This knowledge could be relevant for the development of community strategies to deal with this or other communicable diseases.
